# Larvicidal potential, toxicological assessment, and molecular docking studies of four Egyptian bacterial strains against *Culex pipiens* L. (Diptera: Culicidae)

**DOI:** 10.1038/s41598-023-44279-0

**Published:** 2023-10-11

**Authors:** Tokaa Mansour, Wafaa H. Radwan, Menna Mansour, Mohamed Gomaa, Farouk Farouk, Mohamed Shepl, Ahmed G. Soliman, Basma T. Abd-Elhalim, Mohamed M. K. El-Senosy, Ashraf Bakry, Naglaa M. Ebeed, Neima K. Alsenosy, Hesham Elhariry, Ahmed Galal, Salwa M. El-Sayed, Eslam Adly, Samah H. Abu-Hussien

**Affiliations:** 1 Undergraduate student, Biotechnology Program, Faculty of Agriculture, Ain Shams University, Cairo, 12411 Egypt; 2 Department of Agriculture Microbiology, Faculty of Agriculture, Ain Shams University, Cairo, 12411 Egypt; 3 Department of Plant Protection, Faculty of Agriculture, Ain Shams University, Cairo, 12411 Egypt; 4 Department of Genetics, Faculty of Agriculture, Ain Shams University, Cairo, 12411 Egypt; 5 Department of Food Science, Faculty of Agriculture, Ain Shams University, Cairo, 12411 Egypt; 6 Department of Poultry Production, Faculty of Agriculture, Ain Shams University, Cairo, 12411 Egypt; 7 Department of Agricultural Biochemistry, Faculty of Agriculture, Ain Shams University, Cairo, 12411 Egypt; 8 Department of Entomology, Faculty of Science, Ain Shams University, Cairo, 15611 Egypt

**Keywords:** Biotechnology, Microbiology

## Abstract

Mosquito control in Egypt depends on applying chemical synthetic pesticides that impact negatively on human health and the environment as well as the development of antibiotic and chemical resistance. This study aims to control the 3rd and 4th instars of *Culex pipiens* larvae using four bacterial strains. According to Phenotypic and molecular identification, the four isolates were identified as *Bacillus subtilis MICUL D2023*, *Serratia marcescens MICUL A2023*, *Streptomyces albus LARVICID*, and* Pseudomonas fluorescens MICUL B2023*. All strains were deposited in GenBank under accession numbers OQ764791, OQ729954, OQ726575, and OQ891356, respectively. Larvicidal activity of all microbial strain metabolites against a field strain of *C. pipiens* explored low LC_50_ results and reached its lowest values on the 3rd day with values of 6.40%, 38.4%, and 46.33% for *P. fluorescens*, *S. albus*, and *S. marcescens*, respectively. In addition, metabolites of *P. fluorescence* were more toxic than *those of S. albus*, followed by *S. marcescens. B. subtilis* shows no larvicidal effect on both field and lab mosquito strains. Microscopic alterations of 3rd and 4th instars showed toxic effects on different body parts (thorax, midgut, and anal gills), including losing external hairs, abdominal breakage, and larvae shrinkage, as well as different histological malformations in the digestive tract, midgut, and cortex. GC–MS analysis detected 51, 30, and 32 different active compounds from *S. albus*, *S. marcescens*, and *P. fluorescens*, respectively. GC detected 1, 2-BENZEA2:A52NEDICARBOXYLIC ACID, 2-Cyclohexene-1-carboxylic-acid-5-2-butenyl-methyl ester, and 3 octadecahydro2R3S4Z9Z-11R-12S from *S. albus*, *S. marcesens*, and* P. fluorescens*, respectively. Total protein, Total carbohydrate, and Acetylcholine esterase activity indicated significantly low levels on the 3rd day. All strain metabolites were safe against HSF cell lines. The docking results confirmed the role of the produced metabolites as larvicidal agents and Acetylcholine esterase inhibition. Such a problem need more studies on applying more and more natural pesticides.

## Introduction

Mosquitoes are considered the most dangerous vector for many serious human diseases. The *C. pipiens* mosquito is found in Egypt and spread widely transmitting several viral diseases such as West Nile, Rift Valley, yellow fever, filariasis, and *Wuchereria bancrofti* viruses as well as irritating and bothering humans^1^. Moreover, it is the main cause of various major health issues all over the world. It is well known for causing high mortality and morbidity cases for both animals and humans as well. Also, it is one of the main reasons for heavy economic losses^2^.

The control of mosquitoes in Egypt mostly depends on applying chemical synthetic pesticides as larvicidal and adult-repellent preparations. The misuse of chemical pesticides impact negatively on human health and the environment as well as the development of antibiotic and chemical resistance^3^. Egyptian *C. pipiens* were resistant to insecticides, especially DDT, Permethrin, propoxur, Deltamethrin, Pyrethrum, permethrin (for the control of adult Mosquitoes), organophosphate, and carbamates^4^.

Nowadays, global interest is focused on develop various bioproducts as new alternatives for traditional chemical insecticidal preparations to control mosquitos. Many microbial species have been studied for their larvicidal activities^5^. Bacteria and Actinomycetes include many bacterial and actinomycetal genera which have been characterized for their medicinal and pesticidal potentials. *Streptomyces* sp., *Pseudomonas* sp., *Bacillus* sp., and *Serratia* sp. are microorganisms belonging to bacteria and actinomycetes that have antimicrobial, larvicidal, and pesticidal activities^6^.

Many researchers reported the larvicidal activity of these microorganisms against mosquitoes, especially *C. pipiens*^7^. Additionally, various microbial metabolites are found to be safe for human skin fibroblast^8^. However, a few research studies have reported the role of these metabolites on the acetylcholine esterase activity by molecular docking. So, this study aims to investigate the larvicidal potential of metabolites extracted from *Streptomyces* sp., *Pseudomonas* sp., *Bacillus* sp., and *Serratia* sp. against the 3rd and 4th instars of *C. pipiens* larvae. Moreover, studying their enzymatic activity to illustrate their neurotoxic effect and mode of action using biological and docking assays. Additionally, the determination of bioactive compounds in their produced metabolites using GC–MS.

## Materials and methods

### Chemicals

Acetylcholine esterase enzyme, 5,5-dithiobis (2-nitrobenzoic acid) (DTNB); acetylthiocholine iodide (ATCh); and bovine serum albumin (BSA) were purchased from the Sigma–Aldrich Chemical Co (Taufkirchen, Germany). All chemicals analysis were carried out in Nawa scientific labs. (www.nawah-scientific.com), Mokattam branch, Cairo, Egypt.

### *C. pipiens* larvae collection

According to the guidelines for laboratory and field testing of mosquito larvicides^9^*, C. pipiens* L., larvae samples in various larval instars were collected randomly from October 2022 to January 2023 from three water ponds around three general hospitals (Dar El-Salam, General Hospital, Road El Farag General Hospital and, ElZawya ElHamra General Hospital, Cairo governorate, Egypt. All collected larvae were placed inside sterilized glass jars and transported to the laboratory of the Microbial Inoculant Center, Faculty of Agriculture, Ain Shams University, Cairo, Egypt.

### Microorganisms and media used

Four bacterial isolates (*Bacillus* sp., *Serratia* sp. *Pseudomonas* sp., and *Streptomyces* sp.) were collected from the Microbial Inoculant Center (MIC), Faculty of Agriculture, Ain Shams University, Cairo, Egypt. All media were obtained from Oxoid, UK, where nutrient agar medium was used for subculturing, maintenance and production of larvicidal compounds of *Bacillus* sp. and *Serratia* sp. isolates. Kings B medium was used for subculturing, maintenance, and production of larvicidal compounds of *Pseudomonas* isolate and Starch nitrite medium was used for subculturing, maintenance and production of larvicidal compounds of *Streptomyces* sp. isolate.

### Standard inoculum

Standard inoculum of (1.2 × 10^6^) for bacterial isolates was prepared by inoculating 50mL of the selected medium and incubated at 30 °C for 24 h. For Streptomyces isolate, scratching the agar slants were suspended in 50 mL of Starch nitrite medium and incubated at 39 °C for 7 day.

### Identification of the collected isolates

#### Phenotypic identification

All bacterial isolates were identified according to their cultural and cell morphology characteristics by applying both Gram and spore staining.

#### Genotypic identification

Bacterial DNA was extracted and polymerase chain reaction (PCR) was applied for gene sequencing. Partially amplification of extracted 16s rRNA genes was carried out using the universal primers of 27F (5′ AGAGTTTGATCCTGGCTCAG 3′) and 1492R (5′ TACG GCTACCTTGTTACGACTT 3′). Thereafter, the partially amplified 16s rRNA genes were purified using QIA quick gel extraction kit (Qiagen, Germany)^10^. The purified product was then sequenced via Macrogen company (South Korea) using BioEdit version 7.0.4. After reading, clipping, and aggregating sequences, ClusterW version 4.5.1. was used to align all obtained genetic information. The nucleotide FASTA sequence was submitted to the NCBI GenBank to conduct Blast inquiries and Mega X software was used to build and construct neighbor-joining phylogenetic cladogram. https://www.megasoftware.net/.

### Extraction of the larvicidal products

The produced standard inoculum for all tested strains was inoculated at the suitable medium and incubated at 30 °C for 24 h, 7 days^11^ for bacterial and *Streptomyces* sp., respectively. After incubation, fifteen milliliters of the cultures were collected and centrifuged at 15000 rpm for 20 min. pellets were discarded and the supernatant was collected for the larvicidal assay. All trials were carried out in triplicates.

### Biological larvicidal activity of the identified bacterial strains

All tested culture supernatants of the four bacterial strains were evaluated against the 3rd and 4th instar larvae of both lab and field mosquito strains using the dipping technique^9^ performed in single-use cups containing 100mL of culture supernatants. *Bacillus* sp., *Pseudomonas* sp., *Streptomyces* sp., and *Serratia* sp. culture supernatants were tested at concentrations of 0, 20, 40, 60, 80, and 100% in distilled water (dilutions of 0:5, 1:4, 2:3, 3:2, 4:1 and 5:0 culture supernatant/distilled water). Each bioassay was conducted in three replicates using 25 of the 3rd and 4th instar larvae in each replicate. The total number of dead larvae was recorded after 24 h, 48 h, and 72 h of exposure, respectively. The results of this experiment presented as larval mortality percentage (LMP%) for each treatment. According to the previous results, The LC_50_, slope, and regression factor values were calculated for *Pseudomonas* sp., *Streptomyces* sp., and *Serrtaia* according to the method of Finney^22^, Based on Abbott's formula, the corrected mortality percentage was calculated as follows:

$$Corrected \, mortality \left(\%\right)=\frac{\mathrm{\%test \, mortality}-\mathrm{\% control \, mortality}}{100-\%control \, mortality} \times 100$$

### Morphological, histopathological, and behavioral studies of *Culex pipiens*

Based on the previous results, Hoyer's media was prepared to test the morphological and behavioral alterations of the treated larvae d using a Labomed microscope (at 40x). Briefly, Both the control and treated larvae were fixed in 10% formalin, dried out using ethyl alcohol series, washed in xylene, fixed in paraplast, and cut into sections (7 mm). using staining protocols, the sectioned larvae and control samples were stained with eosin and hematoxylin, respectively. Using a microscope (Labomed), the mid-guts of control and treated larvae were examined and captured^12^.

### Gas chromatography-mass spectrometry (GC/MS) analysis

According to the previous results, *Pseudomonas, Streptomyces*, and *Serratia *sp. supernatants were examined for the produced metabolites^13^. Shortly, culture supernatant was added to ethyl acetate solvent at a ratio cf 1:1 (v/v) and then shaked for 20 min. separation and concentration of ethyl acetate phase was done by evaporating to dryness at 50 °C. purification of the produced residue was carried out using methanol^13^. Compound separation was started by injecting all samples into a capillary column TG-5MS with dimensions 30 m × 0.25 mm × 0.25 m). Trace GC-TSQ mass spectrometer (Thermo Scientific's, Austin, TX, USA) was used. The column temperature was kept at 50 °C for 2 min. before being raised by 5 °C/min. to 250 °C. Expanded at a rate of 30 °C/min. to a maximum temperature of 300 °C and held for 2 min. Expanded at a rate of 30 °C/min. to a maximum temp. of 300 °C and held for 2 min. The carrier gas was helium, with a constant flow rate of 1 mL/min. The injector and MS transfer line temperatures were kept at 270 °C and 260 °C, respectively. With a solvent delay of 4 min, the autosampler AS1300 connected with the GC in split mode automatically injected diluted specimens of 1 μL. In full scan mode, EI mass spectra were acquired at 70 eV ionisation voltages spanning the m/z 50–650 range. The temperature of the ion source was set to 200 °C. The constituents were identified by comparing their mass spectra to those of the WILEY 09 and NIST 14 mass spectral databases^13^.

### Total protein content

Proteins were determined using Pierce™ BCA Protein Assay Kit (Product No. 23225 or 23227) (ThermoScientific). An amount of 50 μL of each protein standard (including a blank) and the sample was transferred to a microcentrifuge tube, Then, 450 μL of distilled water, 100 μL of the sodium deoxycholate (0.15%), and 100 μL of trichloroacetic acid (TCA) solution (72%) were added. The mixture was incubated for 10 min. at room temperature, followed by centrifugation for 15 min at 10,000 rpm. The supernatant was discarded. 50 μL of SDS reagent (5% SDS (w/v) in 0.1 N sodium hydroxide was added to the pellets. In the end, 1 mL of BCA reagent was added and then incubated for 30 min. at 37 °C. Absorbance at 562 nm. Was read. Protein content was calculated as the amount of total protein per 25 larvae^14^.

### Total carbohydrate content

For quantification of total carbohydrates, the Glucose standard for total soluble carbohydrates assay was carried out^14^. Briefly, a glucose stock solution of 1 mg was dissolved in 1 mL of distilled water, and different concentrations (400, 200, 100, 50, 25, and 12.5 µg/mL) were prepared. Larvae samples were homogenized using a sterilized mortar and then centrifuged at 10,000 rpm for 15 min. Pellets were discarded and the supernatant was collected and diluted by a ratio of 1:1 in distilled water. Fifty µL of larvae sample were transferred to a glass vial and mixed with 100 µL concentrated sulfuric acid solution (75% v/v). Then, 200 µL of anthrone reagent (5 mg in 100 µL ethanol and 2.4 mL 75% v/v sulfuric acid) was added and the vial was incubated in an oven with temperature adjusted at 100 °C for 5 min. After heating, the mixture was allowed to cool at room temperature for 5 min. and the analysis was carried out by transferring 100 µL of the sample mixture to a 96 wells plate (n = 6, three independent experiments), where the resulting green color was measured at 578 nm. Data are represented as means ± SD. The results were recorded using a microplate reader^14^.

### Acetylcholine esterase (AChE) activity

A standard solution containing donepezil was prepared at a concentration of 5 mM as a positive control. The acetylcholine esterase inhibitor assay was carried out. Briefly, 10 µL of the indicator solution (0.4 mM in buffer (1): 100 mM tris buffer pH 7.5) was transferred to a 96-well plate followed by 20 µL of enzyme solution (acetylcholine esterase 0.02 U/mL final concentration in buffer (2): 50 mM tris buffer pH 7.5 containing 0.1% bovine serum albumin). 20 µL of the sample/standard solution was added followed by 140 µL of buffer and the mixture was left for 15 min. at room temperature. Afterward, 10 µL of the substrate (0.4 mM acetylcholine iodide buffer was added immediately to all wells. The plate was incubated in a dark chamber for 20 min. at room temperature. At the end of the incubation period, the color was measured at 412 nm. Data are represented as means ± SD. The results were recorded using a microplate^14^.

### Cytotoxicity of the identified strains

Cytotoxicity was carried out for *Pseudomonas*, *Streptomyces*, and *Serratia* strains**.** Cytotoxicity test was carried out in Nawah Scientific Inc. (www.nawah-scientific.com), (Cairo, Egypt). Cell viability was assessed using an SRB assay^15^. Cell viability was expresses as control cell viability (%), which was set as 100%^10^.

### Molecular docking

The three-dimensional model structure of the receptor Acetylcholinesterase from *Culex pipiens (house mosquito)* was retrieved from the UniProt KB database using its ID (Q86GC8). The protein structure was refined using ModRefiner^16^, (https://zhanggroup.org/ModRefiner/). The receptor's active site and potential pockets were identified using Deepsite to predict protein–ligand binding sites^17^ (https://www.playmolecule.com/deepsite/(. The Deepsite results were retrieved from the PlayMolecule platform. All ligands' SMILES and SDF (structure data files) were retrieved from the PubChem database (https://pubchem.ncbi.nlm.nih.gov/). The 3D chemical structures of the ligands were then energy-minimized using Avogadro 1.2.0 software^18^. For protein preparation and docking simulation, The receptor was prepared using AutoDock Tools 1.5.6 version^19^. Hydrogens were added, charges were assigned, and grid box centers were determined. Then, all files were saved as pdbqt files in a separate directory. Docking simulations were performed using AutoDock Vina software^20^, with a grid box size of 20 × 20 × 20 and the following dimensions: center_x = − 5.035, center_y = 16.888, center_z = − 15.678^21^.

### Statistical analysis

The LC_50_, slope, and regression factor values were calculated for *Pseudomonas*, *Streptomyces*, and *Serratia* strains^22^ using the computer program Sigma Plot for Windows, Version 2.0, based on Abbott's formula corrected mortality percentage^23^. Statistical analysis of the experimental data was performed using the computer software SAS 2005 version and MS EXCEL 2016. A Tukey test at a P-value < 0.05 was applied.

### Ethical statement

This article was approved by the Ethics Committee of the Faculty of Agriculture, Ain Shams University, Cairo, Egypt. Also, the research does not contain any studies with human participants or animals performed by any of the authors.

## Results

### Identification of the collected isolates

#### Phenotypic identification

**Figure 1:**
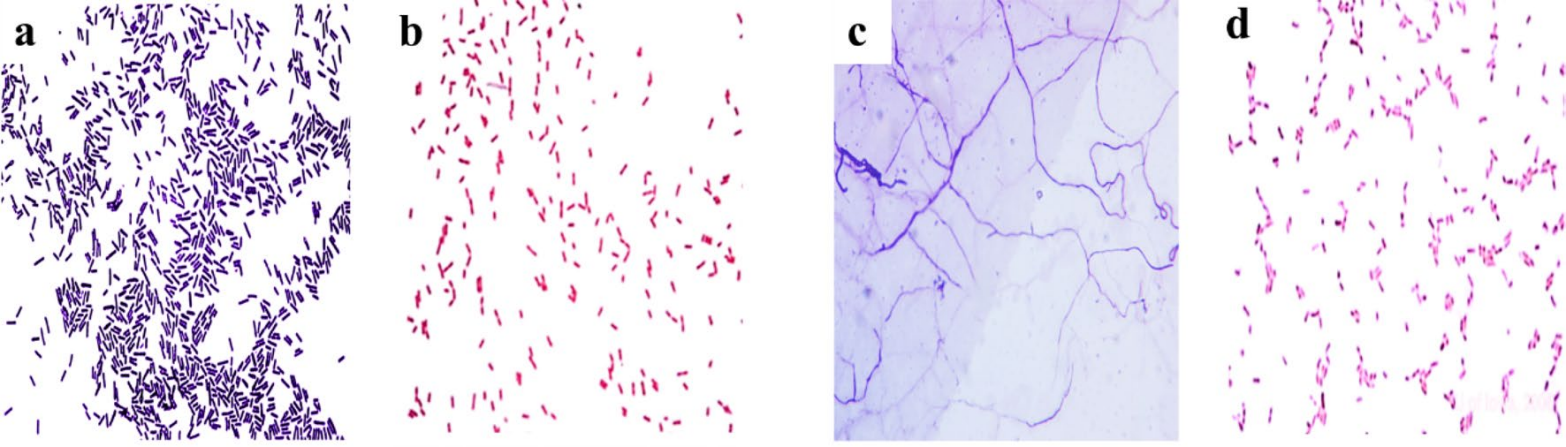
Phenotypic characteristics of collected isolates (**a**) cultural characteristics of *Bacillus* sp. (**b**) cultural characteristics of *Serratia* sp. (**c**) cultural characteristics of *Streptomyces* sp. (**d**) Morphological characteristics of *Pseudomonas* sp.

Phenotypic characteristics of *Bacillus* sp., *Serratia* sp. *Pseudomonas* sp. and *Streptomyces* sp. isolates are illustrated in Fig. 1. *Bacillus* sp. showed white, circular, rough, opaque, fuzzy white, or slightly yellow with jagged edges. They are long rods, sporulated, and Gram + ve. *Serratia* sp. showed red, smooth, convex, entire, and round colonies. They are short rods and Gram-Ve. *Streptomyces* sp. showed white aerial mycelium non-producing melanoid, non-producing soluble pigments. They are bi-branched carrying circular conidiospores and Gram + ve. *Pseudomonas* sp. isolate showed large colonies, irregular surfaces, opaque, and produce green fluorescent pigments. They are small-sized, Gram negative short rods.

#### Molecular identification of the four bacterial isolates

**Figure 2:**
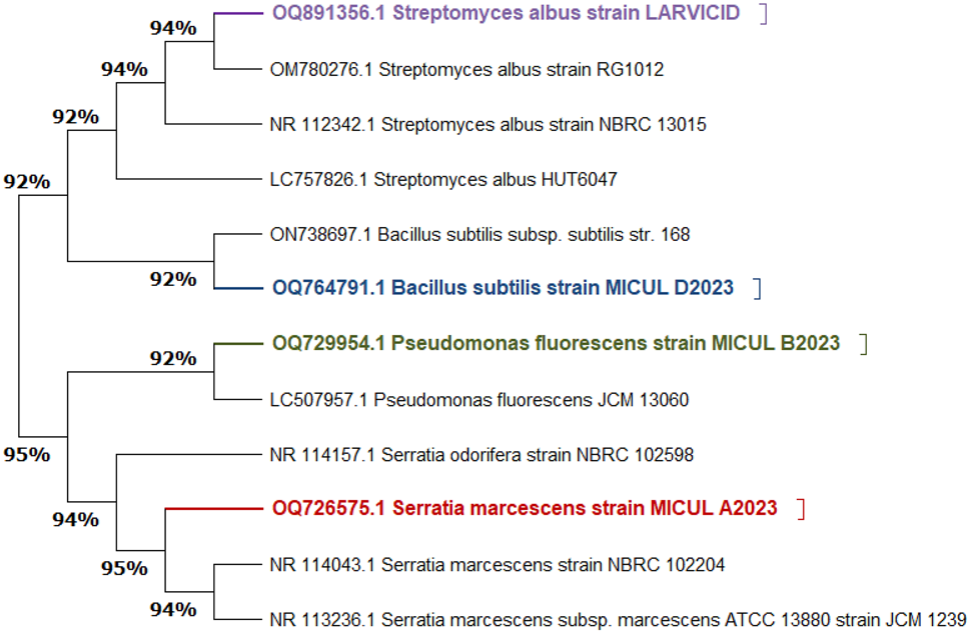
Phylogenetic neighbor‐joining tree based on the 16S rRNA gene sequence of *Bacillus* sp., *Serratia* sp. *Pseudomonas* sp. and *Streptomyces* sp. show a relationship to other strains of phylogenetically close bacterial species. The DNA sequence in the current study was deposited on the GenBank under accession numbers; OQ764791, OQ726575, OQ729954 and OQ891356; respectively.

Results illustrated in Fig. 2. showed that the 16S rRNA gene sequence was similar to *B. subtilis*, *S. marcescens*, *S. albus*, and *P. fluorescens* (NCBI); therefore, it was identified as *B. subtilis MICUL D2023, S, marcescens MICUL A2023, S. albus LARVICID and P. fluorescens MICUL B2023* and deposited in Gen bank unser accession numbers OQ764791, OQ729954, OQ726575, OQ891356, respectively. The similarity between the isolates and their nearest phylogenetic species is shown in Fig. 2**.**, which includes a comparison of the sequence information for the four isolates and their neighbors. The findings revealed 95%, 95%, 94%, and 92% similarity for *Serratia*, *Pseudomonas*, *Streptomyces*, and *Bacillus* neighbors, respectively.

### Biological larvicidal activity of the identified bacterial strains

The mortality percentages of 3rd and 4th instar larvae of field mosquito strains caused by serial (gradual) concentrates of the four bacterial strains. The larvicidal potential was started after 2 days of exposure time, reaching 100% of larvae mortality at the end of 3 days. Larvicidal activity of all microbial strains metabolites against field strain of *C. pipines* was shown in Table 1. After 48 h of exposure, The LC_50_, slope, and regression factor for *P. fluorescens* were 9.81%, 11.81%, 0.91%, respectively. The LC_50_, slope, and regression factor *S. albus* were 50.44%, 11.66%, and 0.91%, respectively. The LC50, slope, and regression factors were 69.1%, 7.46%, and 0.92%, respectively. After 72 h of exposure, The LC50 for *P. fluorescens*, *S. albus*, and *S. marcescens* had less value when compared to its values in 48 h treatment. According to LC50 results, it reached its lowest values on the third day with values of 6.40%, 38.4%, and 46.33% for *P. fluorescens*, *S. albus*, and *S. marcescens*, respectively. In addition, metabolites of *P. fluorescence* were more toxic than *S. albus* followed by *S. marcescens. Bacillus subtilis* show no larvicidal effect on both field and lab mosquito strains for 3 days of exposure to the bacterial metabolites. *P. fluorescens* results indicated 100% mortality for all mosquito strains from day 1 at all concentrations used.

**Table 1:** Larvicidal activity of all microbial strains metabolites against field strain of *C. pipines* after 48 and 72 h of exposure time.

Exposure time (h)	Microbial strain	Conc. (%)	Mortality (%)	LC50	R^2^	X^2^ (df)*	F value	LSD	Intercept	Slope
After 48 h	*P. fluorescens*	0	0	9.81	0.94	8.73	33.27209	1.58	− 11.7364	11.8107
		5	5							
		10	33.3							
		15	80.55							
		20	99							
	*S. albus*	0	0	50.44	0.94	11.68	50.615	2.86	− 19.87	11.65
		20	1							
		40	33.3							
		60	50.55							
		80	80							
		100	99							
	*S. marcesens*	0	0	69.1	0.92	5.071936	34.6885	5.68	− 13.72	7.46
		20	1							
		40	33.33							
		60	33.33							
		80	66.67							
		100	66.67							
After 72 h	*P. fluorescens*	0	0	6.40	0.93	7.21	27.90	2.12	− 8.99	11.08
		5	25							
		10	85							
		15	99.5							
		20	99.9							
	*S. albus*	0	0	38.4	0.95	11.64	64.1	4.10	− 18.01	11.36
		20	6.67							
		40	33.33							
		60	85							
		80	99							
		100	99							
	*S. marcesens*	0	0	46.33	0.82	10.58	14.24	4.33	9.86	− 16.43
		20	6.67							
		40	20							
		60	33.33							
		80	93.33							
		100	99							

*X^2^ (df = 3) for *P. fluorescens* and X^2^ (df = 4) for other strains. Control (distilled water)—nil mortality. *LC50* lethal concentration that kills 50% of the exposed larvae, *R*^*2*^ regression coefficient.

### Morphological and behavioural studies of *Culex pipiens*

**Figure 3:**
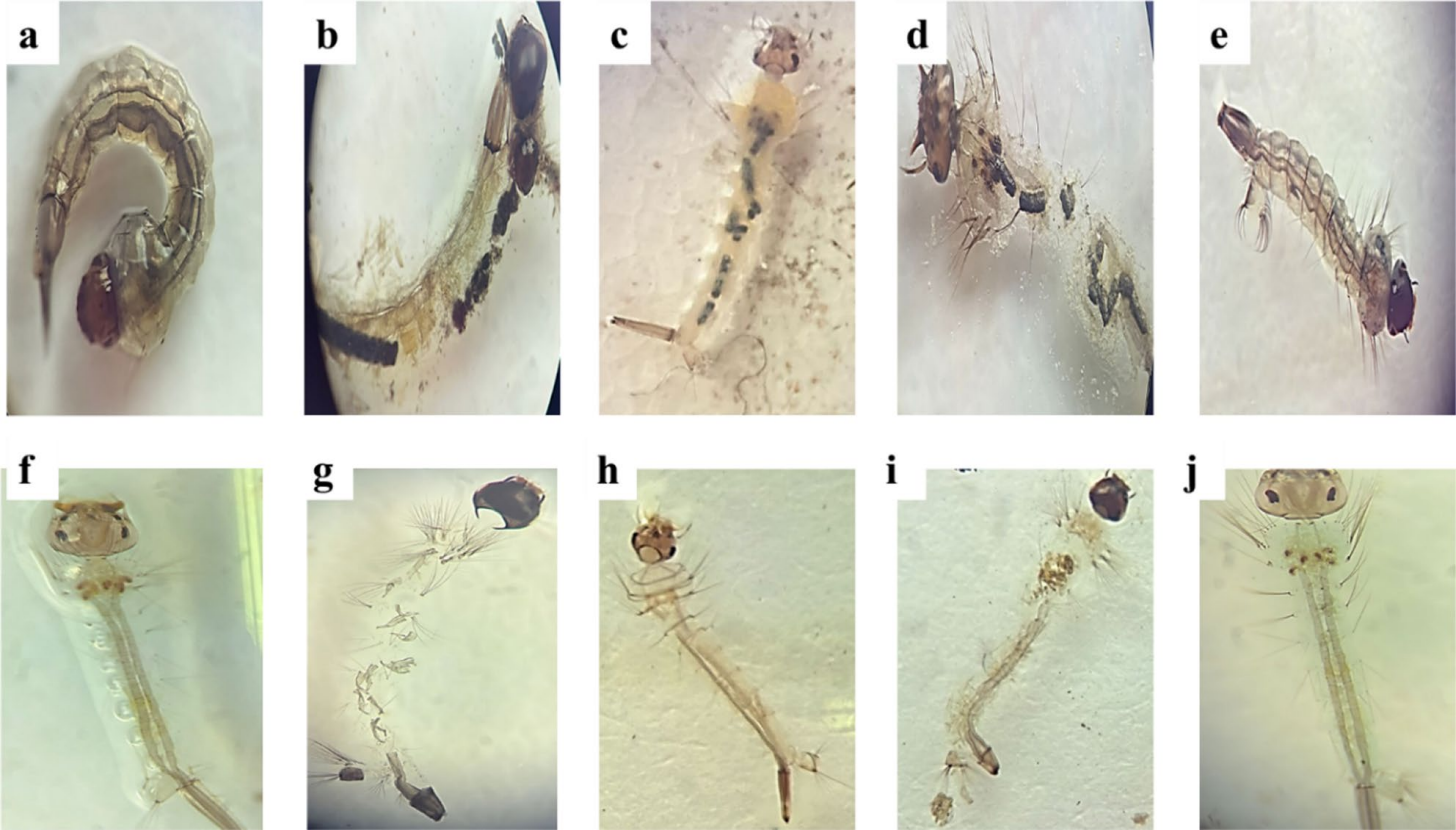
Microscopic alterations of 3rd and 4th instars due to exposure to bacterial metabolites for 72 h (**a**–**e**) field strain, (**f**–**j**) lab strain. (**a**, **f**) control larvae of *C. pipiens* head, thorax, midgut, and anal gill parts. (**b**, **g**) *C. pipiens* was treated with 100% *P. fluores*cence at day 2 (**c**, **h**) *C. pipiens* treated with 100% of *S. marcescens* (**d**, **i**) *C. pipiens* treated with 100% of *S. albus* showing toxic effects on different body parts (thorax, midguts, anal gills) as well as losing external hairs, crumbling outer cuticle of the epithelial layer, abdominal breakage and larvae shrinkage. (**e**, **j**) *C. pipiens* treated with 100% of *B. subtilis* showed no lethal effect on both field and lab strains of *C. pipiens*.

Microscopic alterations of the 3rd and 4th instars due to exposure to bacterial metabolites for 72 h are shown in Fig. 3. *C. pipiens* treated with 100% of *P. fluorescens*, *S. albus*, and *S. marcescens* showed toxic effects on different body parts (thorax, midguts, anal gills) as well as losing external hairs, crumbling outer cuticle of the epithelial layer, abdominal breakage and larvae shrinkage. While *C. pipiens* treated with 100% of *B. subtilis* showed no lethal effect on both field and lab strains.

### Histological changes for *C. pipiens* larvae treated with bacterial metabolites

**Figure 4:**
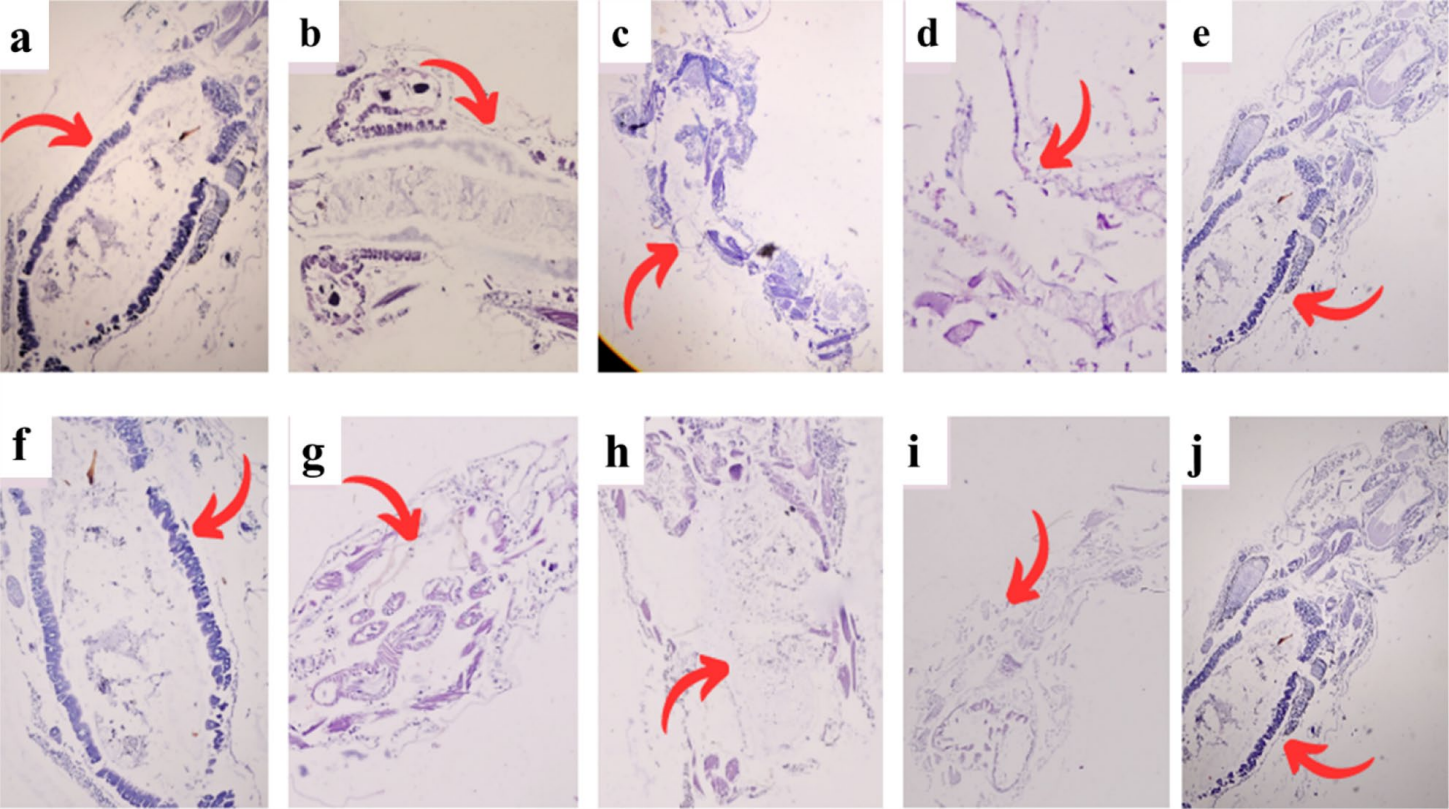
Histopathology malformations for of 3rd and 4th instars due to exposure to bacterial metabolites for 72h (**a**–**e**) field strain, (**f**–**j**) lab strain. (**a**, **f**) transverse section (TS) of control midgut epithelial cells, BM, basement membrane was adherent to epithelial cells, N, spherical nucleus, Mv, or microvilli of the brush border, Pm, peritrophic membrane and GL, gut lumen; (**b**, **g**) *C. pipiens* was treated with 100% *P. fluores*cence at day 2; (**c**, **h**) *C. pipiens* treated with 100% of *S. marcescens*; (**d**, **i**) *C. pipiens* treated with 100% of *S. albus* showing toxic effects on different midgut parts (destruction of epithelial cells, peritrophic membrane and disappearance of microvilli); (**e**, **j**) *C. pipiens* treated with 100% of *B. subtilis* showed no lethal effect on both field and lab strains of *C. pipiens*. Microscopic magnification power (x = 400).

*C. pipiens* treated larvae with 100% bacterial metabolites showed different histological malformations in the digestive tract, midgut, and cortex. These malformations included hyperlapsia for mid-gut epithelial cells, brush border crashing, broken membranes, and cytoplasmic masses existence. The untreated control larvae had both single layers of midgut epithelial cells and digestive cells. Control larvae and the treated *B. subtilis* metabolites were characterized by normal brush border, cell membrane, and cytoplasm as shown in Fig. 4.

### Gas chromatography-mass spectrometry (GC/MS) analysis

According to the results obtained by GC–MS analysis, the main compounds identified in *S. albus* extract were 1, 2-BENZEA2:A52NEDICARBOXYLIC ACID; 1, 3, 5-Triazine-2,4-Diamine; 1-Octen-3-OL-out; 1-Tetradecanol-; 2-Acetyl-3-[2-(cinnamoyl amino)ethyl]-7-methoxy-1H-indole; 2-hydroxy-3-[(9E)-9-octadic; 3-Buten-2-one; Dotriacontane; Estra-1-3-5(10)-trien-17-ol-; Ethyl-cholate; OXIRANEUNDECANOIC-ACID3-PENTYL-METHYL-ESTER; Succindialdehyde and Trilinolein. While the main metabolites produced by *S. marcescens* and identified were 2-Cyclohexene-1-carboxylic-acid-5-2-butenyl-methyl ester; 3,8-Dioxatricyclo-5,1,0,0,2,4-octane-4-ethenyl; 5,6-Epoxy-2,2-dimethyloct-7-ene-3-yne and 6-Octadecenoic acid. On the other hand, Captafol; Captan; Cyclopentadecanone-2-hydroxy, and octadecahydro2R3S4Z9Z-11R-12S were major metabolites extracted from* P. fluorescens* Table 2.

**Table 2:** GC/MS of larvicidal metabolites produced from *S. albus*, *S. marcescens*, and* P. fluorescens.*

Microbial strain	No	RT (min)	Area (%)	Library/ID
*S. albus*	1	4.15	4.7	1,2-BENZEA2:A52NEDICARBOXYLIC ACID
	2	6.29	7.48	1,3,5-TRIAZINE-2–4-DIAMINE
	3	7.06	4.02	1-OCTEN-3-OL-out
	4	13.08	2.27	1-Tetradecanol-
	5	26.18	1.8	2-Acetyl-3-[2_(cinnamoyl amino)ethyl]-7-methoxy-1H-indole
	6	26.96	2.56	2-HYDROXY-3-[(9E)-9-OCTADEC
	7	28.5	0.68	3-Buten-2-one
	8	35.64	3.73	Dotriacontane
	9	35.74	6.04	Estra-1,3,5(10)-trien-17-ol-
	10	36.18	1.31	Ethyl-cholate
	11	7.06	4.02	OXIRANEUNDECANOIC-ACID3-PENTYL-METHYL-ESTER
	12	7.06	4.02	Succindialdehyde
	13	13.08	2.27	Trilinolein
*P. fluorescens*	1	17.854	0.132	Captafol
	2	17.854	0.132	Captan
	3	16.782	0.284	Cyclopentadecanone-2-hydroxy
	4	15.657	0.189	Octadecahydro2R3S4Z9Z-11R-12S
*S. marcesens*	1	17.854	0.132	2-Cyclohexene-1-carboxylic-acid-5-2-butenyl-methyl ester
	2	17.854	0.132	2,7, Bispirocyclopropane bicyclo 22,1heptan
	3	17.854	0.132	5,6-Epoxy-2,2-dimethyloct-7-ene-3-yen
	4	15.657	0.189	6-Octadecenoic acid

### Total protein content

**Figure 5:**
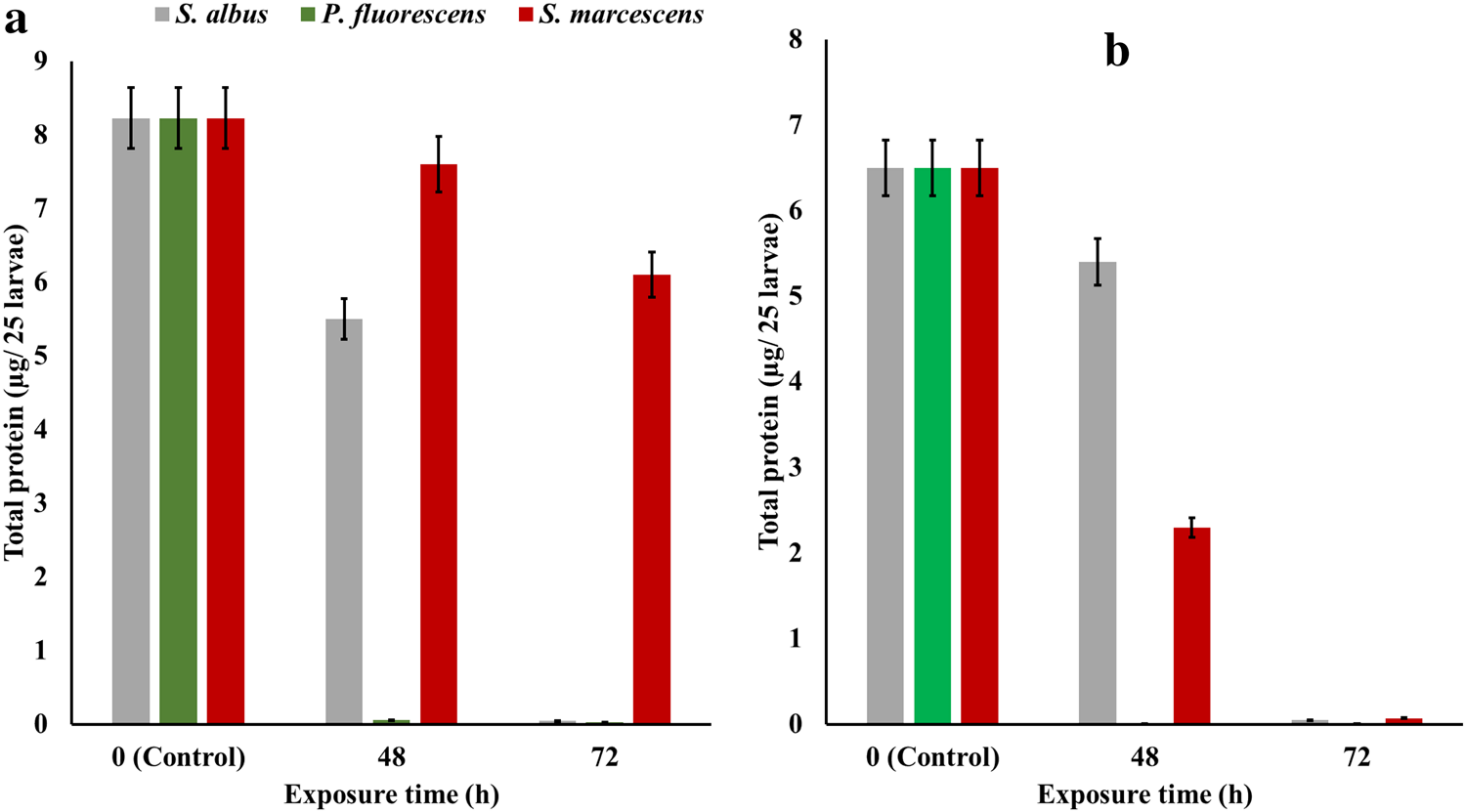
Total protein content (µg/25 larvae) of the 3rd and 4th instar of *C. pipiens* of both field (**a**) and lab (**b**) strains during the exposure for 100% of *S. albus*, *P. fluorescens*, and *S. marcescens* metabolites through 72 h at 25 °C.

Total protein was carried out to observe the chemical changes that occurred in the 3rd and 4th instar of *C. pipines* of both field and lab strains during the exposure for 100% of *S. albus*, *P.fluorescens*, and *S. marcescens* metabolites during 72 h of exposure at 25 °C. In the field strain of *C. pipines* larvae, A significant drop was recorded in the protein content on the third day from 28.23 to 0.05, 0.03and 6.1 µg/25 larvae for *S. albus*, *P. fluorescens*, and *S. marcescens*, respectively. Also, this drop was observed in the lab strain of *C. pipines* larvae after 3 days of exposure to decrease from 6.5 to 0.05, 0.01, and 0.07 µg/25 larvae for *S. albus*, *P. fluorescens*, and *S. marcescens*, respectively Fig. 5.

### Total carbohydrate content

**Figure 6:**
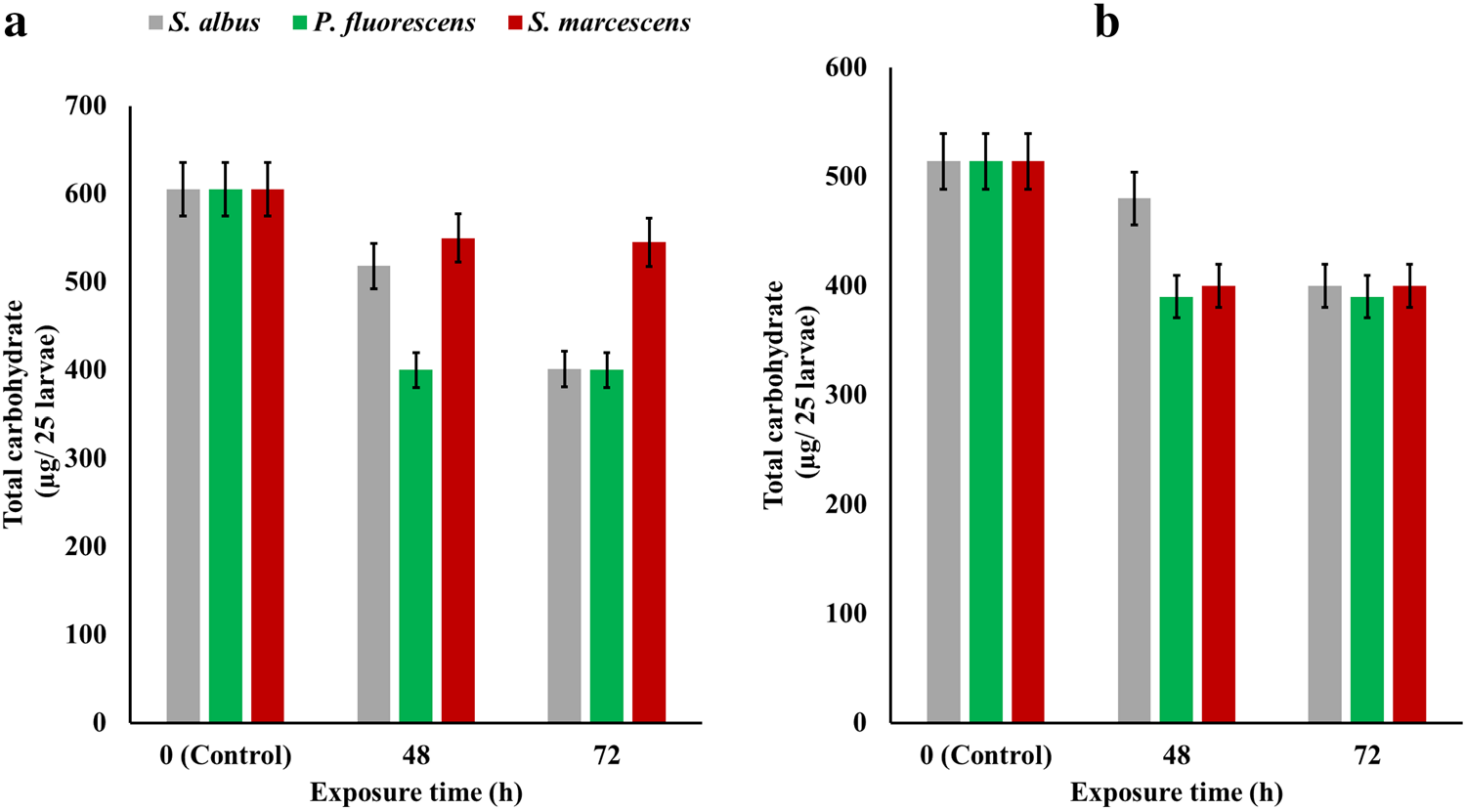
Total carbohydrate content (µg/25 larvae) of the 3rd and 4th instar of *C. pipiens* of both field (**a**) and lab (**b**) strains during the exposure for 100% of *S. albus*, *P. fluorescens*, and *S. marcescens* metabolites through 72 h at 25 °C.

Total carbohydrate was measured in the 3rd and 4th instar of *C. pipiens* of both field and lab strains during the exposure for 100% of *S. albus*, *P.fluorescens* and *S. marcescens* metabolites during 72 h of exposure at 25 °C. In the field strain of *C. pipiens* larvae, A significant drop was recorded in the total carbohydrate content on the third day from 605 to 401, 400, and 545 µg/25 larvae for *S. albus*, *P. fluorescens*, and *S. marcescens*, respectively. Moreover, the lab strain of *C. pipiens* larvae recorded a significant decrease after 3 days of exposure to decrease from 514 to 400, 390, and 400 µg/25 larvae for *S. albus*, *P. fluorescens*, and *S. marcescens*, respectively Fig. 6.

### Acetylcholineesterase activity

**Figure 7:**
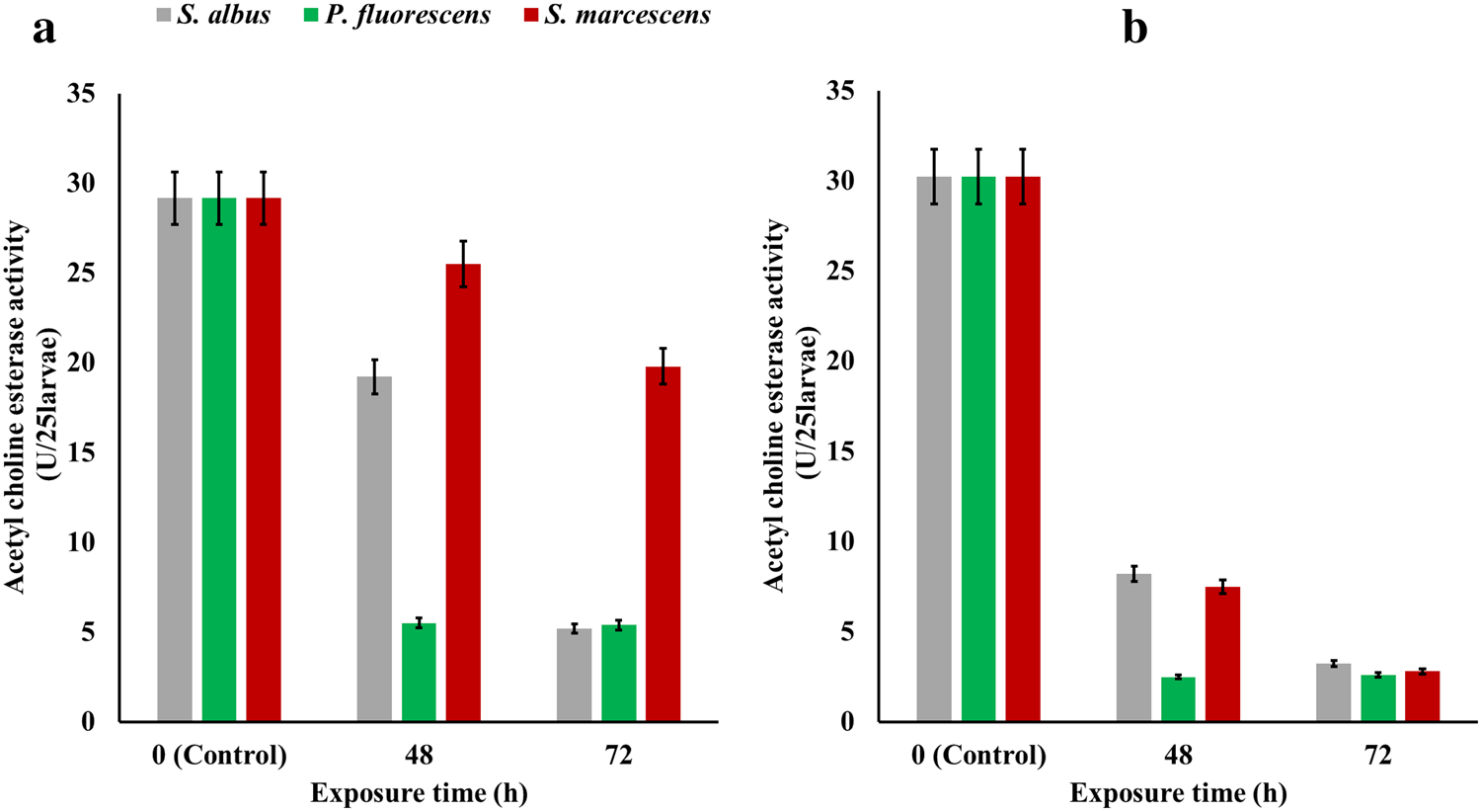
Acetylcholine esterase activity (U/25larvae) of the 3rd and 4th instar of *C. pipiens* of both field (**a**) and lab (**b**) strains during the exposure for 100% of *S. albus*, *P. fluorescens* and *S. marcescens* metabolites through 72 h at 25 °C.

Acetylcholineesterase activity was determined in the 3rd and 4th instar of *C. pipiens* of both field and lab strains during the exposure for 100% of *S. albus*, *P. fluorescens* and *S. marcescens* metabolites during 72 h of exposure at 25 °C. In the field strain of *C. pipiens* larvae, A significantly low activity level was recorded in the total activity after 72 h of exposure from 29.17 to 5.2, 5.4, and 19.8 U/25 larvae for *S. albus*, *P. fluorescens*, and *S. marcescens*, respectively. Also, low levels of acetylcholine esterase activity were observed in the lab strain of *C. pipiens* larvae after 72 h of exposure to decrease from 30.25 to 3.25, 2.6, and 2.8 U/25 larvae for *S. albus*, *P. fluorescens*, and *S. marcescens*, respectively Fig. 7.

### Cytotoxicity of the metabolites produced by *S. albus*, *P. fluorescens*, and *S. marcescens*

**Figure 8:**
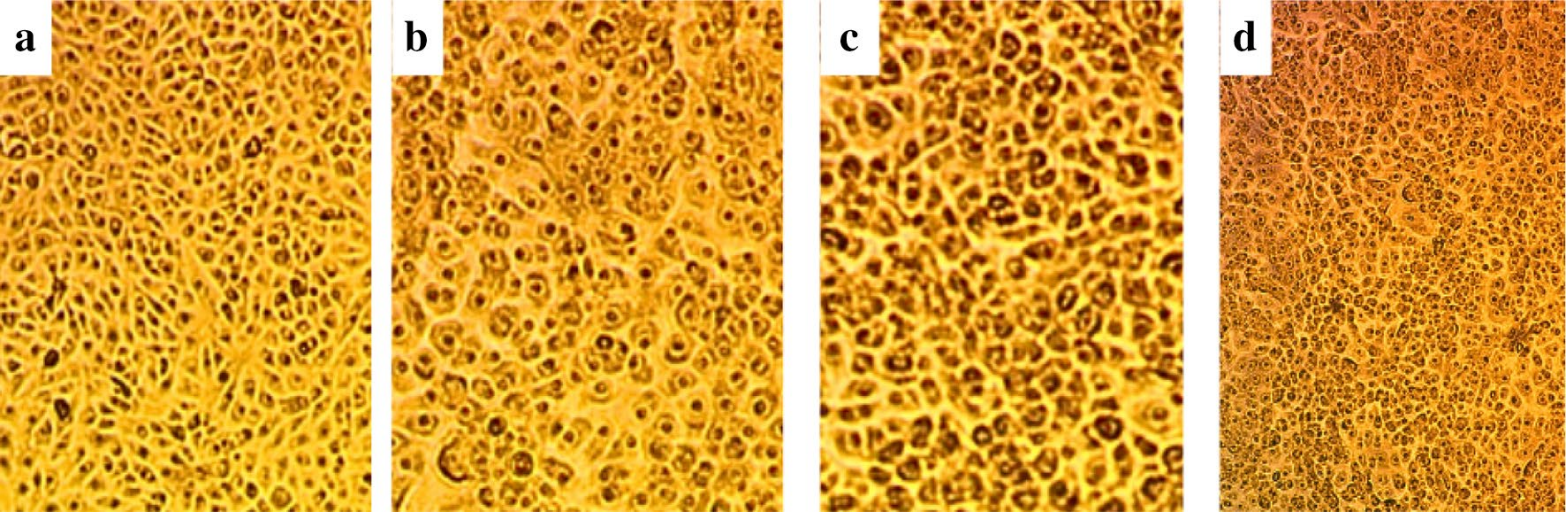
Cytotoxicity of *S. albus*, *P. fluorescens*, and *S. marcescens* metabolites on normal HSF cells maintained in DMEM media supplemented with 100 mg/mL of streptomycin, 100 units/mL of penicillin and 10% of heat-inactivated fetal bovine serum in humidified 5% (v/v) CO_2_ atmosphere incubated at 37 °C (**a**) Control treatment illustrates normal adherent cells; (**b**) *S. albus* metabolites show 95.2% viability; (**c**) *P. fluorescens metabolites* show 95.4% viability; (**d**) *S. marcescens* show 96.1% viability at 100% concentration with few damaged cells illustrated by a reduction in cell adhesion.

*S. albus*, *P. fluorescens*, and *S. marcescens* were tested for cytotoxicity against HSF cell lines using MTT assay at different concentrations of crude extract metabolites from 0 to 100%. Results revealed that HSF cells showed 95.2%, 95.4%, and 96.1% cell viability 100% for the three strains, respectively. Control treatment of HSF cells had 100% viability Fig. 8. All Microscopic images illustrated no significant increase in cytotoxicity for *S. albus*, *P. fluorescens*, and *S. marcescens* when compared to the control treatment.

### Molecular docking

**Figure 9:**
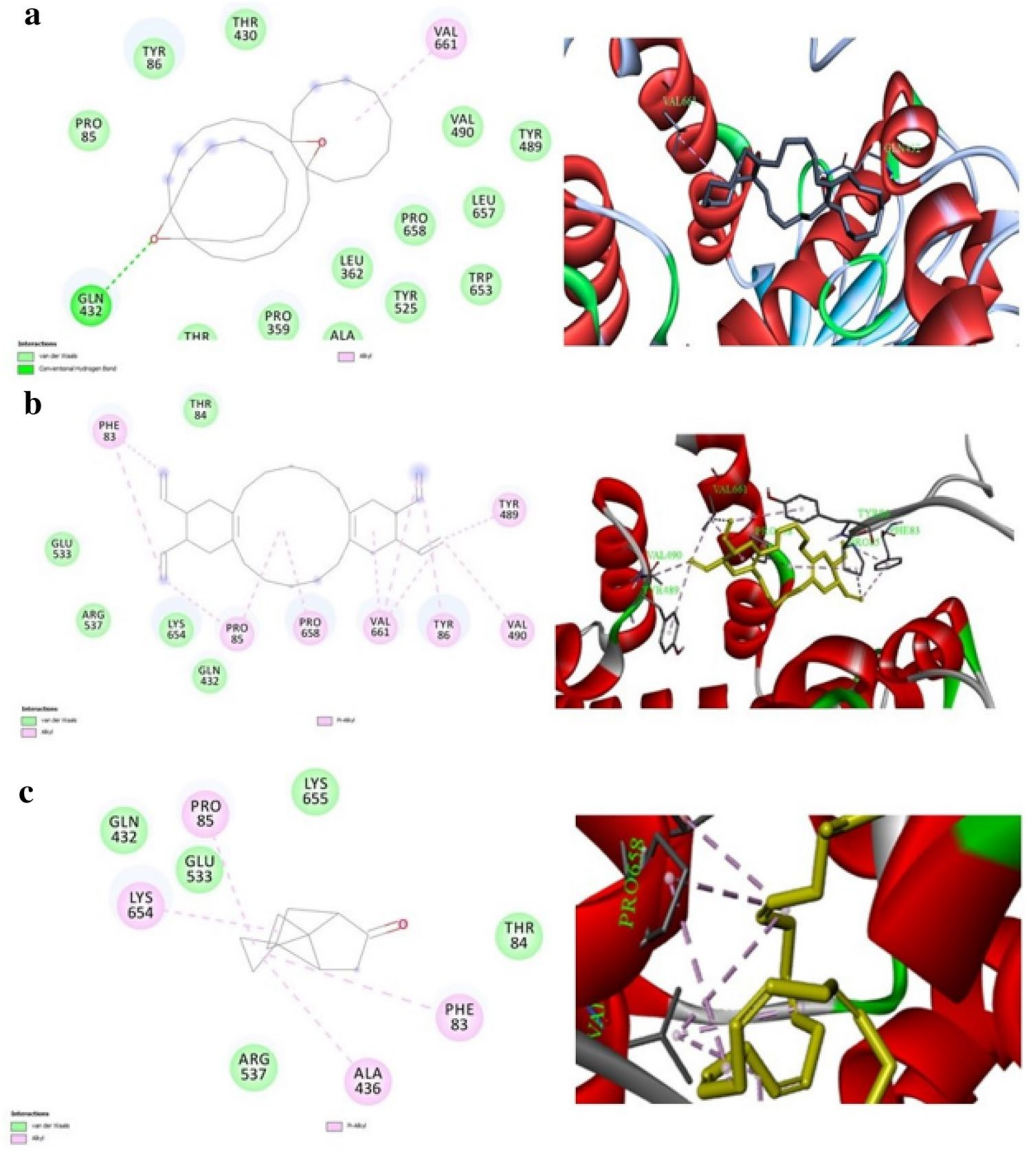
Docking view of Tricyclo 20, 8, 0, 0 (7,16)] triacontane 1(22), 7(16), diepoxy produced by *S. albus* (**a**) Octadecahydro2R3S4Z9Z, 11R, 12S produced by *P. fluorescens* (**b**) and 2,7, Bispirocyclopropane bicyclo 22,1heptan produced by *S. marcescens* (**c**) on the binding sites of AChE (PDB: https://doi.org/10.2210/pdb1EEA/pdb). Left are the 2D interaction diagrams, and right are the complex structures in 3D. All compounds tested were reviewed in the yellow ball and stick-shaped, interaction bonding shown in dashed lines with different colors, the image produced from BIOVIA CLINT 2020.

The docking results suggest that the identified compounds have the potential to inhibit the target protein tested from mosquitoes through different types of interactions. As mentioned in Fig. 9. and Table 3. The compounds from *S. albus* and *S. marcescens* showed hydrogen bonding and alkyl interactions with specific amino acids in the protein, while the compound identified from *P. fluorescens* showed alkyl and pi-alkyl interactions with multiple amino acids. The calculated ΔG values indicate that these interactions are energetically favorable, and the calculated pKd values suggest that the compounds have varying degrees of binding affinity towards the target proteins. The ligand efficiency (LE) values range from 2.61 to 5.49, indicating that the compound identified from *Pseudomonas fluorescens* has the highest potency, followed by the compound from *Streptomyces albus* and then the compound from *Serratia marcescens.*

**Table 3:** Molecular docking and binding interactions of Tricyclo 20, 8, 0, 0 (7,16)] triacontane 1(22), 7(16), diepoxy produced by *S. albus* (**a**) Octadecahydro2R3S4Z9Z, 11R, 12S produced by *P. fluorescens* (**b**) and 2,7, Bispirocyclopropane bicyclo 22,1heptan produced by *S. marcescens* (**c**) on the binding sites of AChE.

Microbial strain	Inhibitor compound	Inhibitor structure	Type of interaction	Interacted AA and ligand atoms	ΔG (Kcal/mol)	pKd	LE	D (Å)^2^
*Streptomyces albus*	Tricyclo 20, 8, 0, 0(7,16)]triacontane 1(22), 7(16), diepoxy		H-Bonding	GLN(432)	− 10	4.99	− 0.21	2.61
			Alkyle interaction	VAL(661)				4.30
*Pseudomonas fluorescens*	Dibenzo[a,h]cyclotetradecene, 2,3,11,12-tetraethenyl-1,2,3,4,5,6,7,8,9,10,11,12,13,14,15,16,17,18-octadecahydro-, (2R*,3S*,4Z,9Z,11R*,12S*)-		Alkyl	PRO(85) PRO(85) PRO(658) VAL(661) VAL(661) VAL(661) VAL(490)	− 9.5	5.19	− 0.23	5.49 4.75 4.09 4.40 5.02 4.17 4.26
			Pi-alkyl	TYR(489) PHE(83) TYR(86)				5.18 5.28 5.14
*Serratia marcescens*	2,7,Bispirocyclopropane bicyclo 22,1heptan		Alkyl	LYS(654) ALA(436) PRO(85)	− 6	2.20	− 0.25	3.99 4.98 4.51
			Pi-alkyl	PHE(83)				5.41

## Discussion

*Culex pipiens* are the main vector for many transmitted diseases like lymphatic filariasis, Rift Valley fever, the bancroftian filariasis and etc., which are true to be controlled. Chemical pesticides have negative hazardous effects on health by elevating insecticide resistance, and non-eco-friendly effects on all living microorganisms. As a result, many research studies help in improving eco-friendly, safe, and natural methods to control *C. pipiens*. Such methods include using various bioactive compounds such as polyphenolic extracts, peptides^24^, nanomaterials ^25^, and essential oils^26^, in addition to using microbial metabolites^27^. Several researchers have developed various types of bio larvicidal agents with effective LC50 and LC90 values against many diseases caused by mosquito-borne vectors.

The present study focused on using natural microbial metabolite products from *S. albus, P. florescens*, and *S. marcescens*. *Streptomyces* species have been considered a repository of a diverse range of secondary metabolism because of their powerful and complex secondary metabolism^28^. Around 100,000 antibiotic compounds are produced by *Streptomyces,* accounting for 70–80% of all natural chemical substances with pharmacological or agrochemical^27,29,30^.

Fifty-one different metabolites extracted from *Streptomyces albus* were detected through the current study. Pathogenicity of *Pseudomonas, Serratia, Streptomyces*, and *Bacillus* species against several insect pests, particularly lepidopteran caterpillars, has recently been documented^31^. These bacteria produce toxins with modes of action similar to *B. thuringiensis*^32^. During bacterial sporulation, *B. thuringiensis* creates a crystal protein (-endotoxin) capable of inducing gut cell lysis when ingested by susceptible insects^33^. Additionally, the extensive paralysis on the midgut epithelium layer might be the major reason for the mortal of *C. pipiens*, in addition to midgut deterioration, dropping the function of midgut and feeding refraining with various microorganisms^34^, *Bacillus subtilis* (MH370499) and *Streptomyces griseoruber* (MH370498)^12^. This deterioration is due to microbial peptides and carbohydrates that increase the pH level (Alkaly) in the insect gut, leading the insects to lose the ability to grow and reproduction^35^. It is generally acknowledged that insects exposed to these products may come into exposure to hazardous substances that affect a variety of Proteins (including enzymes, receptors, signaling molecules, ion channels, and structural proteins) as well as nucleic acids, biomembranes, and other cell components^24^.

Acetylcholinesterase (AChE) is an enzyme that plays a key role in the synaptic transmission of nerve impulses in living organisms. It composes of carbamate and organophosphorus^36^. Cholinesterase inhibition is carried out by using growth regulators^37^ and pesticides resulting in cholinergic toxicity. Pesticides containing carbamate or phosphate were discovered to disrupt the equilibrium between acetylcholine synthesis and its release and its hydrolysis causing synaptic acetylcholine accumulation, resulting in prolonged activation of cholinergic receptors. Secondary metabolites from microorganisms and plants have been found to reduce AchE activity. The *Choristoneura rosacea* larvae treated with neem oil showed a dose-dependent response of -carboxylesterase activity^38^.

According to the present study, metabolites Secreted by *S. albus, P. florescens,* and *S. marcescens* inhibit the AchE enzyme from functioning in *Culex pipiens*, and the inhibition of AChE enzymes by extracts of secondary metabolites was dependent on the type of these compounds leading to the inhibition of AChE enzymes and ultimate death of the organism. Because the mechanism of the larvicidal potential of *S. albus*, *P. fluorescens*, and *S. marcescens* metabolites is not wholly understood, Molecular docking is a method used for identifying and understanding molecular recognition. It identifies the major binding mode and binding affinity between protein and ligand and provides a 3D structural explanation for the interaction in a variety of biological processes^39^. To better focus on the compounds of highly efficient larvicidal potential according to molecular docking analysis. Our results indicated that *S. albus, P. fluorescens*, and *S. marcescens* metabolites affected the activity of AChE compared with the control. This may be attributed to the binding of Tricyclo 20, 8, 0, 0 (7,16)]triacontane 1(22), 7(16), diepoxy produced by *S. albus* with the binding sites of AChE by a hydrogen bond in the residue GlN 432 and an alkyl interaction in the residue VAL 66.

In addition to, Dibenzo[a,h]cyclotetradecene, 2,3,11,12-tetraethenyl-1,2,3,4,5,6,7,8,9,10,11,12,13,14,15,16,17,18-octadecahydro-, (2R*,3S*,4Z,9Z,11R*,12S*)-extracted from *P. fluorescens* is binding with the catalytic residues PRO(85), PRO(85), PRO(658), VAL(661), VAL(661), and VAL(490) at the active site of the enzyme by Alkyle interactions. While, 2,7, Bispirocyclopropane bicyclo produced by *S. marcescens* on the binding sites of AChE by alkyl interactions in the residue LYS(654), ALA(436), and PRO(85). *S. albus, P. fluorescens*, and *S. marcescens* metabolites have a low binding energy and a high affinity for the functional pocket of the AChE target, and they can attach to the active sites of ACHE enzyme, according to the docking study. The presence of bioactive metabolites in bacteria under study provides hope for the development of new natural insecticides that would be economically and environmentally sound for the management of insect pests that affect stored products.

## Conclusion

In conclusion, metabolites of *S. marcescens*, *S. albus*, and *P. fluorescens* had high larvicidal activity against the 3rd and 4th larval instars of *C. pipiens*. On the other hand, *B. subtilis* didn’t show any larvicidal activity. *P. fluooresence* produced the superior larvicidal metabolites followed by *S. albus* and *S. marcescens*, respectively. All produced larvicidal metabolites were safe to humans. Molecular docking confirmed the role of produced metabolites in the inhibition of Acetylcholine esterase and larval mortality.

## Data Availability

The data that support the findings of this study are available from the corresponding author upon reasonable request. Raw sequencing files and associated metadata have been deposited at NCBI's Sequence Read Archive: *Bacillus subtilis* MICUL D2023 https://www.ncbi.nlm.nih.gov/nuccore/OQ764791, *Pseudomonas fluorescens* MICUL B2023 https://www.ncbi.nlm.nih.gov/nuccore/OQ729954, *Serratia marcescens* MICUL A2023 https://www.ncbi.nlm.nih.gov/nuccore/OQ726575, *Streptomyces albus* Larvicid https://www.ncbi.nlm.nih.gov/nuccore/OQ891356, Acetylcholine esterase PDB: PDB: https://doi.org/10.2210/pdb1EEA/pdb.
